# “Unveiling the genetic symphony: Diversity and expression of chicken *IFITM* genes in Aseel and Kadaknath breeds”

**DOI:** 10.1016/j.heliyon.2024.e37729

**Published:** 2024-09-10

**Authors:** Malarmathi Muthusamy, Murali Nagarajan, Sivakumar Karuppusamy, Kannaki T. Ramasamy, Amutha Ramasamy, Ramya Kalaivanan, Gopala Krishna Murthy Thippicettipalayam Ramasamy, Thiruvenkadan Aranganoor Kannan

**Affiliations:** a Veterinary College and Research Institute, Tamil Nadu Veterinary and Animal Sciences University, Namakkal, 637 002, India; b Alambadi Cattle Breed Research Centre, Tamil Nadu Veterinary and Animal Sciences University, Dharmapuri, 635 111, India; c Faculty of Food and Agriculture, The University of the West Indies, St Augustine, Trinidad and Tobago; d ICAR-Directorate of Poultry Research, Hyderabad, India; e Poultry Disease Diagnosis and Surveillance Laboratory, Veterinary College and Research Institute Campus, Tamil Nadu Veterinary and Animal Sciences University, Namakkal, India

**Keywords:** *chIFITM*, *IR-IFITM*, *IFN* γ, *Mx*, Aseel, Kadaknath, SNVs, Chicken

## Abstract

In this investigation, single nucleotide variants (SNVs) within the chicken interferon-inducible transmembrane protein (*chIFITM*) genes were explored in Aseel and Kadaknath breeds. Comparative analysis with the GRCg6a reference genome revealed 9 and 16 SNVs in the *chIFITM* locus for Aseel and Kadaknath breeds, respectively. When referencing the Genome Reference Consortium GRCg7b, Kadaknath exhibited 10 variants, contrasting with none in Aseel. Notably, 17, 8, 2, and 5 SNVs were identified in *chIFITM1*, *chIFITM2*, *chIFITM3*, and *chIFITM5* genes, with *chIFITM1* showing the highest polymorphism in Kadaknath, featuring 10 intronic variants, including three SNVs (rs16457112, rs16457111, and rs313341707) common to both breeds. Two synonymous exonic variants (g.1817767C > A and g.1819102C > T) were also noted in chIFITM1. Although *chIFITM* protein sequences were generally conserved, genetic variations clustered predominantly in UTR and intronic regions. Examination of immune response dynamics in live embryos uncovered notable variations in *chIFITM* gene expression across diverse organs and chicken breeds. Specifically, *chIFITM1* mRNA was abundant in cecal tonsils for both breeds and bursa of Aseel (7.61 folds), but it was absent in the heart and lung tissues of both breeds. Conversely, *chIFITM3* consistently exhibited heightened expression, particularly in bursa of Aseel (10.23 folds). Whereas mRNA of the *chIFITM2* gene was found to be abundant in the heart of Kadaknath (11.03 folds) and lung of both breeds. Furthermore, the expression pattern of *chIFITM5* diverged between the two breeds, the heart of Kadaknath chickens showed highest (10.45 folds). The study discovered that breed-specific genetic variants within these genes present a potential pathway for selection and breeding to improve disease resistance in chicken. The observed genetic variation among chicken populations highlights the critical importance of these variants in reinforcing virus resistance, exhibiting applicability across a wide range of breeds.

## Highlights

• The *chIFITM1* gene was found to be highly polymorphic in Kadaknath chicken breed.
• The robust and consistent expression of the *chIFITM3* gene across various tissues substantiates its potent antiviral efficacy against viral infections.
• The coding sequences of all *chIFITM* genes demonstrate a high level of conservation.

## 1 Introduction

Interferon-induced transmembrane protein (*IFITM*) gene family was first discovered as an IFN-stimulable response element (ISRE) in human neuroblastoma cells [1]. They were primarily named as 9–27 (*IFITM1*), 1-8D (*IFITM2*), and 1–8U (*IFITM3*) [2] and found that it has antiviral activity towards various viruses affecting humans, such as vesicular stomatitis virus (Alber and Staeheli 1996), highly pathogenic Influenza A virus, West Nile Virus, Dengue Virus [3], Zika Virus [4]. Besides, it was also found that they had a strong inhibitory effect on the replication by restricting the viral attachment with the cell membrane of animal and avian virus [5]. Recent evolutionary study reports showed that *IFITM* genes were positively selected as potent innate genes [[6], [7], [8]] and showed resistance against various livestock diseases such as African swine fever virus (ASFV) [9], Avian and swine influenza viruses [[10], [11], [12], [13], [14], [15]], Lyssaviruses [11], foot and mouth disease virus [16], Avian reovirus [17], Infectious bronchitis virus [18,19] and Newcastle disease virus [20].

A decade ago, human *IFITM* genes were characterized and found five *IFITM* homologs (*IFITM1*, *IFITM2*, *IFITM3*, *IFITM5*, and *IFITM10*) in the human *IFITM* gene locus [21]. Further, three more—*IFITM*4, *IFITM*6, and *IFITM*9 have been discovered in zebra fish [6]. In addition to *huIFITMs*, *IFITM*s from mice, Swine [15,22,23], Ducks [24], Goose [25] and Canine [26], Chicken [27] have also been studied by various scientists. Recently, re-sequencing was utilized to characterize the *chIFITM* area, and it found that this locus does not appear to have a lot of repetitive sequences or a very high GC content [10].

The chicken *IFITM* gene locus is surrounded by the genes for the centromeric Acid Trehalase-Like 1 (*ATHL1*) gene and the telomeric β −1,4-N Acetyl-Galactosaminyl Transferase 4 (*B4GALNT4*) in the q arm of Chromosome 5 of its genome [6,11,27,28]. These two genes are highly conserved among species, from mammals to amphibians [11]. Relatively high levels of sequence homology (41.9–67.2 per cent) were found between the chicken and human *IFITM* genes [10]. The chicken *IFITM* family comprises five members of the gene, and they were grouped as immunity-related *IFITM* (*IFITM1*, *IFITM2*, and *IFITM3*) and *IFITM5* and *IFITM10* were exerts indistinct immune function [29,30]. The vertebrate *IFITM* genes are typically found in two loci, one containing the *IFITM10* gene and the other having *IFITM5* and *IR*-*IFITM* (*IFITM* 1, 2, 3) genes [6,27,28]. The *IFITM5* gene encodes a membrane protein that is well-known to play a role in the formation, osteoblast maturation and mineralization of bones [5,22] and it emerged from the bony fish species. The goal of *IFITM10* is still not understood and it was revealed that it may be restricted to the tetrapod species [5,6,11,29]. Furthermore, it was discovered that the *chIFITM2* and *chIFITM1* loci are oriented oppositely compared to their human counterparts. Also, the transcriptional units of the *chIFITM1*,2, 3, and 5 genes have a consistent orientation [27]. It has been found that the *IR*-*chIFITM* genes located at chromosome 5q are in the following order: centromeric - B4GALNT4 - *chIFITM3* - *chIFITM2* - *chIFITM1* -*chIFITM5* - ATHL1 – telomeric [28]. The chicken *IFITM* locus was previously described as *IFITM1* and has been annotated as ‘*chIFITM3*’ and *IFITM3* was called ‘*chIFITM1*’ [11]. All the *chIFITM* genes continue to have their characteristic genetic structure, with two exons separated by a single intron [11,27].

The evolution of *IFITM* genes in Avian species had shown patterns of gene duplication and positive selection [28]. Particularly in the *IR-IFITM* sub-family, which had shown many duplications specific to certain species and lineages. Considering the long interaction of vertebrates with viral infections and positive selection, suggests that the *IR-IFITM* gene variations could be linked to defense mechanisms [6]. Complementing this perspective, more recent studies have indicated that the SNVs rs12252 and rs6598045 within the *huIFITM3* gene are significantly associated with the intensity of IAV infections and the death rates from COVID-19 [[31], [32], [33], [34], [35], [36]]. Further, subtle changes in the *IFITM3* gene correlate with higher susceptibility to ulcerative colitis [37] and also a raised risk of developing hemorrhagic fever with renal syndrome [38,39]. Concurrently, specific SNPs in the *chIFITM1* and *3* genes are closely related to the susceptibility of chickens to the H7N9 virus [32]. Consistent with other ISGs, it is suggested that genetic variations might be associated with infection susceptibility or severity both within and between species. While variations in *IFITM* have been correlated with differential susceptibility to infections in various bird species [40], a thorough investigation into the *IFITM* locus genetic variations, especially in Indian chicken breeds like Aseel and Kadaknath, has yet to be carried out. These chicken breeds are reared in the backyard, which have adapted to their native environment, including weather, endemic diseases, and feeding preferences. As a result, they may have distinct and continuous positive selection for their immunity, illness resistance, and tolerance to tropical environments [41]. Immunological responses of Aseel, Kadaknath, and White Leghorn chickens to sheep RBS cells and NDV demonstrated that Aseel and Kadaknath chickens had greater immunological reactivity than White Leghorn chicks [20,42]. However, there is no research on the *IFITM* gene in India's native chickens like Aseel and Kadaknath, hence this study has been made for analyzing the SNV variations of the *IR-chIFITM* genes in Aseel and Kadaknath chicken breeds. It is essential to uncover genetic alterations that might be highlighted significant differences between these two breeds.

## 2 Materials and methods

### 2.1 Experimental birds and sample collection

A total of 72 blood samples (36 Aseel and 36 Kadaknath) were collected from different poultry flocks *viz.,* Poultry Farm, Veterinary College and Research Institute, Namakkal; Regional Research and Education Centre, Pudukkottai, and local poultry farmer's flock (Dindigul, Karur and Mohanur) of Tamil Nadu, South India. The genomic DNA from the blood samples of Aseel and Kadaknath was extracted using DNeasy Blood and Tissue Kit (Qiagen, Germany Cat. No. 69504) as per the manufacturer's protocol. The genomic DNA samples of both breeds were pooled into two pools per breed (18 samples per pool) [43].

Sixty (30 Aseel and 30 Kadaknath) SPF chicken embryos, 19 days old, were acquired from the Department of Poultry Science at VCRI in Namakkal and utilized in the viral challenge study. With the exception of a control group of 15 Aseel and 15 Kadaknath, the remaining 15 Aseel and 15 Kadaknath embryos were exposed to a velogenic strain of Newcastle disease virus (NDV) at a dosage of 50 percent Embryo Infective Dose (105 EID50). Subsequently, these embryos were maintained in an egg incubator at a temperature of 38 °C and a relative humidity range of 65–75 percent until tissue harvesting at various hours after infection.

### 2.2 Primer designing

Primers for *chIFITM* genes were designed for PCR amplification of *chIFITM1, 2, 3* and *5* genes, and also for real time qPCR amplification of targeted genes (Table 1). Primers were designed based on the Chicken (taxid:9031) sequences (NC_052536.1) available in the NCBI by using the online primer designing tool *Primer-NCBI BLAST*/Primer 3 software (https://www.ncbi.nlm.nih.gov/tools/primer-blast/). A total of 10 g of pooled genomic DNA per sample was utilized for PCR to amplify the complete *chIFITM1, 2, 3* and *5* genes, which ranged between 1.8 and 2.1 kb in size.

**Table 1:** Primers designed for amplification of *chIFITM* genes.

Gene Name	Forward/Reverse	Sequence (5′-3′)	Primer length (bp)	Amplicon Size (bp)
*chIFITM1*	Forward	TATCCTTTACACCACAGCTGGC	22	1799
	Reverse	TTGTCCAATCACCTTGTCCTAGC	23	
*chIFITM2*	Forward	CAGCTAGGACAAGGTGATTGGAC	23	2173
	Reverse	ATGGCTAGAGAAGCATGGAACTT	23	
*chIFITM3*	Forward	AACATCCAGGCTCACCGCTA	20	1764
	Reverse	TCACCCATCCGGGAGCTTAG	20	
*chIFITM5*	Forward	ACTGCCTTACCAGACATCTTCAG	23	1928
	Reverse	ACACATTTGGCTGGACTAGATGA	23	

### 2.3 Sequencing and SNV prediction

The Illumina deep sequencing method was used to sequence the samples. The samples have been sequenced with 15× coverage with an average read length of 150bp. Sequence reads were analyzed for quality by FastaQC and low-quality reads were trimmed using Trimmomatic [45]. The mean phred score value for the sequence quality across each base position (150bp) in the read ranges from 36.29 to 33.31. After trimming, the sequences were stored in fastaQ format for further analysis [46]. All sequences were submitted to the Sequence Read Archive (SRA) of NCBI in FASTQ format, and they were assigned with the following accession numbers i.e., SRR24893278 and SRR24793329 for the Aseel female and male samples, while SRR24881115 and SRR24881091 for the Kadaknath female and male samples. The trimmed sequences were mapped with the reference genome [45,46] The reference genome Red Jungle fowl (GRCg6a) [47] and Cross of Broiler mother x White Leghorn layer father (*GRCg7b*) [48] were used to assess and categorize the location and type of consequence predicted for each SNV. The results were visualized using the Integrated Genome Browser [49]. SAMtools 0.1.19 and BCFtools [50,51] managed the final variant calling, producing a combined VCF for all samples [45,52,53]. All SNVs were screened in the VCF file (Variant call file) and the SNVs were characterized based on location (Intronic, Exon, upstream, downstream, splice site, 5′ UTR, or 3’ UTR).

### 2.4 Validation of *chIFITM* genes with gene-specific primers

The *chIFITM* genes were amplified to validate the gene using specifically designed primers in a Thermal Cycler (Bio-Rad). A total reaction volume of PCR mixture was 25 μL, with 1 μL DNA template, 1 μL of each primer (10 pM), 12.5 μL Ampliqon Taq 2× PCR Master mix (comprising 1.5 mM MgCl_2_, Taq DNA polymerase, and 100 μM dNTPs), and 9.5 μL nuclease-free water. The amplification condition was set with initial denaturation at 95 °C for 3 min; 35 cycles of denaturation at 95 °C for 30 s, annealing at 57.5 °C/56.5 °C/63.6 °C/62.5 °C for 30 s (based on the specific primer set in use), and extension at 72 °C for 1.5 min and the final extension at 72 °C for 5 min. The PCR products were evaluated using 2 % agarose gel electrophoresis. The complete gene size of DNA fragments of *chIFITM1, 2, 3* and *5* genes were amplified, and the corresponding PCR product sizes were 1799, 2173, 1764 and 1928 bp respectively.

### 2.5 *In ovo* gene expression profiling

Various organ tissues such as the Lung, Heart, Liver, Intestine and Bursa of Fabricius were collected from NDV infected and control Aseel and Kadaknath chicken embryos at 3, 6, 12, 24 and 48 hpi. Tissues were immediately stored in RNAlater® (ThermoFisher) at −80 °C until RNA isolation. The total RNA was extracted by Trizole method using RNAiso Plus, M/s Takara, (Cat. No: 9109). The relative expression of specific mRNA was quantified [54] by a real-time thermal cycler (Roche LightCycler® 96). Gene expression studies, include all the four targeted genes (*chIFITM*1, 2, 3 and 5) alone with two positive immune-related genes (*IFN*- γ and *Mx*) were assessed and β-actin was the gene used as the reference gene. The Mx gene is included as positive immune gene against viruses and also it was an Interferon Stimulating Gene family. The quantitative Real-time PCR (qRT-PCR) was performed to quantify the targeted genes using the designed primers (Table 2) and Bio-Rad Univer SYBR green Master mix (Cat.No: 1725271). The real-time PCR cycling conditions were, initial denaturation at 95 °C for 10 min followed by 40 cycles of denaturation at 95 °C for 10 min, followed by 40 cycles of 95 °C for 10s and 60 °C–62 °C (*β* Actin, *IFN γ*, *Mx, chIFITM (1, 2, 3 and 5*) for 45s and 72 °C for 15s. For each, the sample dissociation curve (melt curve) was generated after the completion of amplification. A negative control containing all ingredients except the cDNA template (non-template control; NTC) was set up invariably for each master mix made for conducting the reactions. Each sample was run in duplicate. Transcript levels were normalized to those for the chicken housekeeping gene β-actin. The alteration in mRNA levels of each gene in NDV-infected embryos was quantified as a fold change using the 2^−ΔΔCt^ method [55], in comparison to the non-infected mock control.

**Table 2:** List of primer sequences for qRT-PCR.

Gene	Primer	Sequence (5'--3′)	Reference
*chIFITM1*	Forward	GCAGGATGTGACCACCACTA	NM_001350059.2
	Reverse	CTTCGCTGTCCTCCCATAGC	
*chIFITM*2	Forward	AACAGGCGGAGGTGAGCAT	NM_001350058.2
	Reverse	AAGATGAGCGAGGGGAAGCA	
*chIFITM3*	Forward	CGTGAAGTCCAGGGATCGCA	NM_001350061.2
	Reverse	GCAACCAGGGCGATGATGAG	
*chIFITM*5	Forward	CCAACCCCACTTCTGGACGA	NM_001199498.1
	Reverse	ATCACTCCGAAGGGCACGAC	
*chMx*	Forward	GTCCAAGAGGCTGAATAACAGAG	NM_204609
	Reverse	GTCGGATCTTTCTGTCATATTGG	
ch*IFN*-γ	Forward	TGAGCCAGATTGTTTCGATG	[44]
	Reverse	CTTGGCCAGGTCCATGATA	
*chβ*-*Actin*	Forward	TATGTGCAAGGCCGGTTTC	
	Reverse	TGTCTTTCTGGCCCATACCAA	

### 2.6 Prediction of *chIFITM* proteins and comparison

The potential open reading frames (ORFs) of the respective *chIFITM* genes were predicted by ORF finder in NCBI database (https://www.ncbi.nlm.nih.gov/gorf/gorf.html). Based on this, the corresponding coding sequences for Aseel and Kadaknath IFITM genes were taken. The DNA sequences of these breeds were subsequently translated into their peptide sequences using the EMBOSS Transeq tool available online (https://www.ebi.ac.uk/Tools/st/emboss_transeq/). A comprehensive alignment of these translated amino acid sequences, benchmarked against the reference chicken *IFITM* protein sequence sourced from the Uniprot database, was executed using Kalign ClustalW [12].

### 2.7 Statistical analysis

Gene expression was assessed in five distinct tissues at five distinct time points in each of the two breeds. The time points in each breed's tissue were 3, 6, 12, 24, and 48 h following infection. ANOVA was used to analyze the significance of the mRNA expression levels within breeds and confirm any variation in time intervals within breeds. The significance of the gene expression level at various times in each tissue between breeds was later analyzed using the *t*-test because only two breeds were compared. All analyses were performed using the R program (version 4.2), with a p-value <0.05 considered statistically significant. Graphic illustrations were also created using R.

## 3 Results

The sequences were evaluated after being deep sequenced using Illumina-based NGS technology platforms. The preliminary analysis targeted the *chIFITM* locus, identifying the presence of variants and their distribution in each *chIFITM* genes of the respective chicken breeds relative to the reference chicken genome (Table 3). The results showed the presence of nine and sixteen SNVs across the *chIFITM* locus in Aseel and Kadaknath breeds, respectively with respective to the reference genome Red Jungle fowl (GRCg6a) across coding and non-coding regions of the locus. In contrast, using the Cross of Broiler mother x White Leghorn layer father (*GRCg7b)* as the reference genome, the Kadaknath chicken breed exhibited ten variants in the *chIFITM* locus and for the Aseel breed, no SNVs were identified.

**Table 3:** Number of SNVs in *chIFITMs* gene as per the reference genomes.

Gene Symbol	*GRCg6a*		*GRCg7b*	
	Aseel	Kadaknath	Aseel	Kadaknath
*chIFITM 1*	3	14	–	3
*chIFITM 2*	4	–	–	4
*chIFITM 3*	1	1	–	–
*chIFITM 5*	1	1	–	3
Total number of SNVs	9	16	–	10

### 3.1 *IR-IFITM* gene variants

Based on the reference sequences from the Genome Reference Consortium (*GRCg6a* and *GRCg7b*) accessible in the NCBI, the coding and non-coding regions of the three antiviral *IR-chIFITM and chIFITM5* (two exons and one intron per *IFITM*) genes were investigated for nucleotide variation.

The *chIFITM1* gene was located in the locus LOC422993 of chromosome number 5:g.1817672-1819250bp position and found 14 SNVs in the region of the *chIFITM1* gene with respect to the Genome Reference Consortium *GRCg6a* (Table 4). This comprised 10 SNVs in the intronic region, three of which were present in both Aseel and Kadaknath breeds, whereas the remaining seven were only discovered in the Kadaknath *chIFITM1* gene. In the exonic region of the *chIFITM1* gene in Kadaknath, single nucleotide variations were discovered, i.e., g.1817767C > A and g.1819102C > T in Exon 1 and 2, respectively, whereas g.1819135A > G and g.1819157C > T were present in three prime untranslated region (3’ UTR) region of Exon 2. These SNVs were confirmed to be available in the Ensembl genome database.

**Table 4:** SNVs of *chIFITM1* as per the Red Jungle fowl reference genome (GRCg6a).

Position as per *GRCg6a*	Reference Allele	Alternate Allele	Variation Type	Consequence	Location	Feature Sequence	Existing Variation	Breed
1817767	C	T	SNP	Synonymous variant	EXON 1	NM_001350059.1	rs317414539	Kadaknath
1819102	C	A			EXON 2	NM_001350059.1	rs316752027	Kadaknath
1819135	A	G		3′ UTR variant		NM_001350059.1	rs16457103	Kadaknath
1819157	C	T				NM_001350059.1	rs317795576	Kadaknath
1817999	G	C		Intronic variant	INTRON **1**	ERR7564039.23466	rs16457112	Aseel/Kadaknath
1818006	A	G				ERR7564039.23466	rs16457111	Aseel/Kadaknath
1818213	T	C				ERR7564039.23466	rs313341707	Aseel/Kadaknath
1818260	C	T				ERR7564039.23466	rs313434559	Kadaknath
1818278	A	G				ERR7564039.23466	rs313015881	Kadaknath
1818455	T	C				ERR7564039.23466	rs16457108	Kadaknath
1818462	G	A				ERR7564039.23466	rs314739981	Kadaknath
1818473	A	G				ERR7564039.23466	rs16457107	Kadaknath
1818835	T	C				ERR7564023.10248	rs13649904	Kadaknath
1818841	G	A				ERR7564023.10248	rs16457105	Kadaknath

While the *chIFITM2* gene was positioned next to the *chIFITM1* gene at the locus LOC107053353 in the position g.1819414 - 1821290 of chicken chromosomal number 5, in *chIFITM2* of the Aseel chicken, four distinct nucleotide variances were found in relation to the reference genome *GRCg6a* (Table 5). A complex nucleotide variant was noticed as CCAC > TCAT at five prime untranslated region (5′UTR) of Exon1, which was the combination of two SNPs viz., rs16457102 and rs16457101. Detection of a downstream gene variant at exon 1 of the *chIFITM2* gene revealed multi-nucleotide polymorphism (MNP) as g.1820823CC > TG. This demonstrated the merging of two existing SNPs viz., rs735111122 and rs739838225, in SNPdb. Two A > G transition mutations were identified at the 3′UTR and 5′UTR of exon 1 and 2, respectively, of *chIFITM2*.

**Table 5:** SNVs of *chIFITM2* as per the Red Jungle fowl reference genome (GRCg6a).

Position as per *GRCg6a*	Reference Allele	Alternate Allele	Variation Type	Consequence	Location	Feature Sequence	Existing Variation	Breed
1819423 -1819426	CCAC	TCAT	Complex	5′ UTR variant	EXON 1	NM_001350058.1	rs16457102/rs16457101	Aseel
1820823 -1820824	CC	TG	MNP	3′ UTR variant	EXON 1	ERR7564035.14760	rs735111122/rs739838225	Aseel
1820837	A	G	SNP	3′ UTR variant	EXON 1	ERR7564035.14760	rs732321039	Aseel
1821251	A	G		5′ UTR variant	EXON 2	ERR2368565.61130	rs14508756	Aseel

As per the Genome Reference Consortium *GRCg6a,* the *chIFITM3* gene was located at the LOC770612 locus of chromosome 5g.1814234-1815550, which was flanked at the 5′prime by the *B4GALNT4* genes and the 3′prime by the *chIFITM1* genes. A transition SNP at g.1814938G > A in the 3′UTR of Exon 1 in and a transversion SNP at g.1815386A > C of Exon 2 were found, respectively (Table 6) in Aseel and Kadaknath breeds.

**Table 6:** SNVs of *chIFITM3* as per the Red Jungle fowl reference genome (GRCg6a).

Position as per *GRCg6a*	Reference Allele	Alternate Allele	Variation Type	Consequence	Location	Feature Sequence	Existing Variation	Breed
1814938	**G**	**A**	SNP	3′ UTR variant	EXON **1**	ERR7564031.18410	rs16457122	Aseel
1815386	**A**	**C**	SNP	5′ UTR variant	EXON **2**	ERR7564047.38625	rs10725914	Kadaknath

In comparison to the reference genome of GRCg7b, the *chIFITM1, 2, and 3* were positioned at chromosome 5:g.1776991-1778570, 1778734-1780610 and 1773554 – 1774870 respectively. Three new transition mutations were found in *chIFITM1* exon1. Similarly, four novel nucleotide polymorphisms were observed at LOC107053353 of Kadaknath chicken breed, including two transitions, one multi-nucleotide Variation (MNV) and one INDEL (insertion) (Table 7).

**Table 7:** SNVs in Kadaknath as per the reference genome Cross of Broiler mother x White Leghorn layer father (*GRCg7b*).

Position as per (*GRCg7b*)	Reference Allele	Alternate Allele	Variation Type	Consequence	Gene Symbol	Location	Feature Sequence
1777551	G	A	SNP	3′ UTR variant	*chIFITM1*	EXON 1	ERR7564039.23466
1777587	A	G				EXON 1	ERR7564039.23466
1777972	A	G		5′ UTR variant		EXON 1	ERR7564023.10394
1778830	A	G			*chIFITM2*	EXON 1	NM_001350058.1
1779054	NNNNNNN	TTTTTTT	MNP			EXON 1	NM_001350058.1
1779409	A	AC	INS	Intron variant		INTRON 1	NM_001350058.1
1780160	A	G	SNP	Intron variant		INTRON 1	ERR7564035.14760
1781267	C	T	SNP	5′ UTR variant	*chIFITM5*	EXON 1	NM_001199498.1
1781282	G	A				EXON 1	NM_001199498.1
1781411	A	G				EXON 1	NM_001199498.1

### 3.2 *chIFITM5* gene variants

According to the reference sequence *GRCg6a*, the *chIFITM5* gene was placed after *chIFITM2* and before *ATHL1* at the location of chromosome 5:1821594-1823163 (+). The SNVs were identified at the 5′ UTR variant in Exon 1 of Kadaknath (g. 1821812C > T) and Exon 2 of Aseel (g. 1822662C > G), and upon verification in the Ensemble database, it has been confirmed that two variations were present, each with its own unique reference, *viz.*, SNP cluster IDs of rs3386028259 and rs74110888 (Table 8). As per the reference genome of *GRCg7b,* the *chIFITM5* gene was located at chromosome 5:g.1780914-1782483. Three new transition mutations at 5′UTR of exon 1 were noticed while compared with the reference genome, *GRCg7b*.

**Table 8:** SNVs of *chIFITM5* as per the Red Jungle fowl genome (*GRCg6a*).

Position as per *GRCg6a*	Reference Allele	Alternate Allele	Variation Type	Consequence	Location	Feature Sequence	Existing Variation	Breed
1821812	C	T	SNP	5′ UTR variant	EXON 1	NM_001199498.1	rs3386028259	Kadaknath
1822662	C	G	SNP	5′ UTR variant	EXON 2	SRR13267647.1959	rs741108889	Aseel

### 3.3 Prediction of *IR-chIFITM* protein and comparison

The *chIFITM* genes of Kadaknath and Aseel chickens were analyzed through sequencing, and the results revealed a total of 27 SNVs in *IR-chIFITM* genes, with 17 SNVs in *chIFITM1*, 8 SNVs in *chIFITM2,* and 2 SNVs in *chIFITM3* genes. Six SNVs were found in the *chIFITM5* gene. Most of the SNVs were found in the UTR regions and introns of *chIFITM* genes in both breeds. Two SNVs were specifically located in the open reading frame (ORF) of the Kadaknath *chIFITM1* gene, resulting in a change of codons. Further analysis (Table 9) indicated that these exonic variations led to synonymous mutations.

**Table 9:** Predicted consequences of SNVs in Kadaknath as per the *GRCg6a*.

Symbol	Genomic position	cDNA position	CDS position	Protein position	Amino acids	Codons Ref/Alt	Existing variation
*chIFITM1*	1817767	96	42	14	S	tcC/tcT	rs317414539
	1819102	388	334	112	R	Cgg/Agg	rs316752027

### 3.4 *In ovo chIFITM* gene expression against NDV

We conducted an *in-ovo* ND viral challenging investigation as a continuation of the *in-vitro* viral challenging study to gain a deeper understanding of the tissue-specific chIFITM gene expression patterns. There was a significant difference in immune gene expression (Table 10) between breeds (p < 0.05), genes (p < 0.01), and tissues (p < 0.0001). On the other hand, the interactions among various factors profoundly influenced immune gene expression (p < 0.01).

**Table 10:** Analysis of Variance (ANOVA) of immune gene expression in chicken embryo against NDV.

Source of variance	Df	SS	MS	F value	Pr(>F)	Significance
Breed	1	5.9	5.85	6.356	0.01193	*
Gene	5	105.5	21.1	22.916	<2e-16	***
Tissue	4	206.3	51.58	56.013	<2e-16	***
Time point: Breed	5	14.5	2.9	3.145	0.00815	**
Time point: Gene	25	176.7	7.07	7.676	<2e-16	***
Breed: Gene	5	634.3	126.87	137.761	<2e-16	***
Time point: Tissue	20	177.2	8.86	9.62	<2e-16	***
Breed: Tissue	4	618.4	154.6	167.879	<2e-16	***
Gene: Tissue	18	1742	96.78	105.09	<2e-16	***
Time point: Breed: Gene	25	320.5	12.82	13.921	<2e-16	***
Time point: Breed: Tissue	20	252.5	12.62	13.707	<2e-16	***
Time point: Gene: Tissue	90	1014	11.27	12.234	<2e-16	***
Breed: Gene: Tissue	18	831.1	46.17	50.139	<2e-16	***
Time point: Breed: Gene: Tissue	90	929.8	10.33	11.219	<2e-16	***
Residuals	672	618.9	0.92			

*p < 0.05; **p < 0.01; ***p < 0.001.

The average fold change of *IR*-*chIFITM* gene expression in response to NDV across different tissues, including the bursa, cecal tonsils, heart, lung, and spleen was calculated. There were significant differences noted in the expression of immune genes within breeds at various post-infection time points across all tissues in Aseel and Kadaknath. Similarly, disparities among breeds in terms of transcript levels of immune genes at different post-infection stages were noticed.

#### 3.4.1 *IR*-*chIFITM* gene expression

The study involved an analysis of the relative quantification of mRNA levels for three *IR-chIFITM* genes, namely *chIFITM1*, *chIFITM2*, and *chIFITM3*, in various tissues of infected chicken embryos from two different breeds, Aseel and Kadaknath, at different time points post-infection (hpi). In the bursal tissue, all three *IR*-*chIFITM* genes were significantly up-regulated in Aseel, with the most pronounced expression observed for *chIFITM1* and *chIFITM3* up to 24 and 6 hpi and showed highest fold changes as of 7.61 and 10.23, respectively. Meanwhile, *chIFITM2* exhibited a moderate up-regulation, with the highest fold change of 2.92 at 48 hpi. Significant upregulation was detected in Kadaknath; however, it was less prominent than in Aseel, with the maximum expression of *chIFITM1* at 6 hpi (2.79 folds) and 24 hpi (2.49 folds), *chIFITM2* at 3 hpi (3.29 folds) and 6 hpi (2.30 folds), and *chIFITM3* at 3 hpi (3.86 folds) to 24 hpi (4.15 folds), was observed respectively.

In cecal tonsil tissue, *chIFITM1* showed consistent upregulation in Aseel, peaking at 12 hpi with a fold change of 9.47. A mild upregulation was observed for *chIFITM2* at 3 hpi, while significant upregulation was noticed at 3 (2.97 folds) and 6 hpi (2.54 folds) for *chIFITM3*. In Kadaknath, strong and significant upregulation was observed throughout the study period for *chIFITM1*, with the highest fold change ranging from 2.13 to 8.67. c*hIFITM2* exhibited significant upregulation at 3 and 6 hpi with the fold change of 2.11 nd 2.42 folds, respectively. While *chIFITM3* mRNA was abundant only at 24 hpi (5.69 folds).

In embryonic heart tissue, the *chIFITM1* gene expression was undetectable in both breeds. *Throughout the study, chIFITM2 showed significant and stable upregulation in both Aseel and Kadaknath. chIFITM3* also displayed stable upregulation across all time points in both breeds (2.08–3.29 folds). In lung tissue, *chIFITM1* exhibited strong upregulation at 6 hpi (6.05 folds), 12 hpi (2.40 folds) and 24 hpi (3.12 folds) in Aseel. Whereas strong upregulation was noticed at 24 hpi (4.22 folds) and 48 hpi (6.17 folds) in Kadaknath. The *chIFITM2* had significant upregulation at 24 hpi (6.40 folds) in Aseel. At the same time, abundant mRNA of *chIFITM2* was found throughout the post-infection period in Kadaknath and it ranged from 5.23 to 17.91 folds. The *chIFITM3* showed stable upregulation was noticed as 3.09–4.10 folds throughout the period of study in Aseel. Contrary to Aseel *chIFITM3* gene expression was until 12 hpi in Kadaknath as 3.17 folds.

In spleen tissue, *chIFITM1* gene expression was undetectable in both Aseel and Kadaknath. In Aseel, *chIFITM2* displayed significant upregulation from 3 hpi to 12 hpi, with the highest expression as 3.51 folds. Similarly, in Kadaknath, *chIFITM2* was significantly upregulated from 3 to 24 hpi, with the highest expression at 4.08 folds. The *chIFITM3* showed stable expression throughout the period of study in Aseel, with a range of 2.09–3.53 folds, while in Kadaknath, it exhibited mild to moderate expression, with a range of 1.70–2.18 folds.

#### 3.4.2 *chIFITM5* gene expression

The gene expression profiling of the *chIFITM5* gene was carried out in all five tissue samples in both chicken breeds. The results demonstrated significant (*p < 0.01*) upregulation of the *chIFITM5* gene in chicken embryos at all time points, except in lung tissue. Specifically, in Aseel, the highest fold change of *chIFITM5* gene expression was 8.70, 9.79, and 7.42 folds at 3 hpi in bursa, cecal tonsil, and spleen, respectively. While, at 24 hpi a fold change of 7.80 folds was noticed in heart tissue. Strong upregulation was observed at 24 hpi (6.02 folds) in the lung. In Kadaknath, the results showed a significant (p < 0.01) upregulation of the *chIFITM5* gene in chicken embryos at all time points only in heart tissue. The highest fold change was noticed at 3 hpi, and 48 hpi and the same was maintained as 8.37–10.45 folds throughout the time points.

#### 3.4.3 *IFN-γ* and *Mx* gene expression

The expression profiling of *IFN*-*γ* and *Mx* genes was included as immune positive genes. The results showed that the *IFN*-*γ* gene was significantly and consistently upregulated in most of the tissue samples in both breeds. The *IFN*-*γ* gene exhibited peak levels of 8.90 (6 hpi), 4.35 (6 hpi), 2.17 (24 hpi) 2.75 (3 hpi), and 2.47 (48 hpi), respectively in bursa, cecal tonsil, heart, lung and spleen in Aseel breed. While in Kadaknath, the peak levels were 2.76 (3 hpi), 9.36 (12 hpi), 5.49 (6 hpi), 2.51 (48 hpi) and 3.00 (3 hpi), respectively. The gene expression fold changes for the *Mx* gene were observed in Aseel chickens, with the highest expression at 24 hpi (6.23 folds), 3 hpi (2.27 folds), 12 hpi (2.62 folds), 12 hpi (4.25 folds), and 48 hpi (3.26 folds), respectively, in bursa, cecal tonsil, heart, lung, and spleen. While in Kadaknath, the highest expression was observed at 24 hpi (10.68 folds), 24 hpi (6.75 folds), 6 hpi (4.86 folds), 24 hpi (6.95 folds), and 3 hpi (7.77 folds), respectively.

**Fig. 1:**
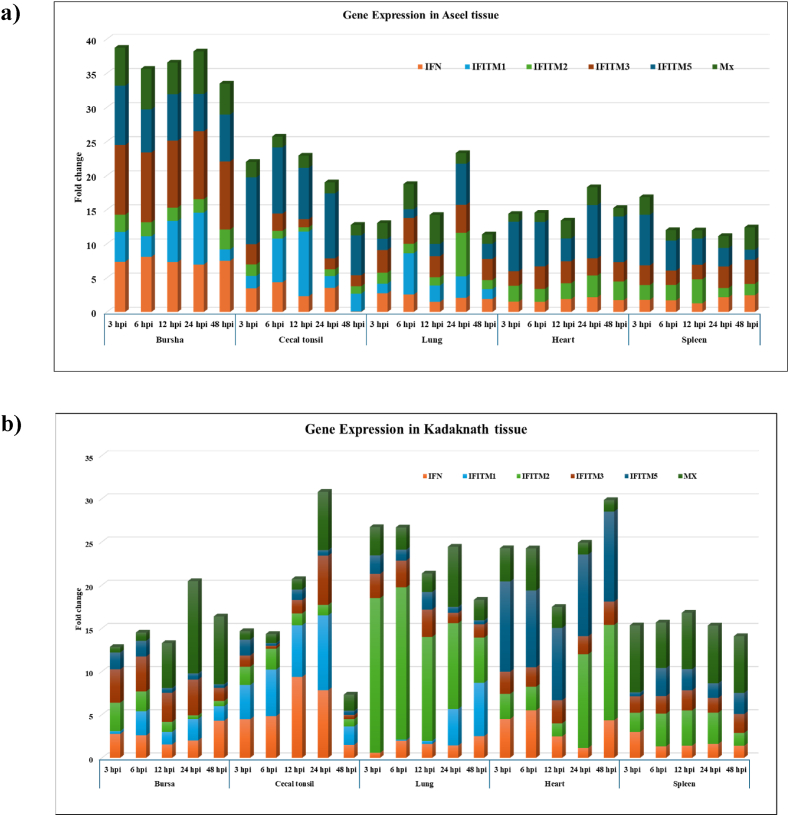
Expression levels of immune genes in embryonic tissues of (a) Aseel and (b) Kadaknath.

**Fig. 2:**
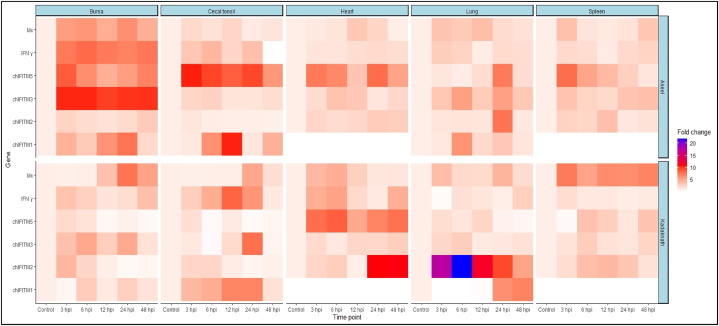
Heatmap illustrating tissue specific and breed specific immune gene expression patterns against NDV infection in Aseel and Kadaknath chicken embryos.

Fig. 1(a and b) shows marked variations in the expression of immune genes within breeds at various post-infection time points across all tissues in Aseel and Kadaknath. Fig. 2 depicts the heat map underscoring the notable disparities among breeds in terms of immune gene transcript levels at different post-infection stages.

## 4 Discussion

### 4.1 Characterization of chicken *IFITM* (*chIFITM*) locus

The *chIFITM* locus was found to be flanked by the *ATHL1* and *B4GALNT4* genes [27,28]. The identified SNVs were located in both coding and non-coding regions of the locus. Similarly, it was found that chicken cell lines such as HD11, DF1, and OU-2 cells were a total of 27 SNVs identified in the coding and noncoding regions of the *chIFITM* gene based on *Gallus gallus*_5 reference genome, indicating relatively low sequence diversity or variation [10]. Whereas a limited number of *IFITM* gene variations were present in a total of 206 samples from different chicken breeds, with 10, 15, 7, and 18 SNVs, respectively, in the European, Commercial, Pirbright, and local chicken breeds [28]. The findings highlighted the genetic diversity present in the *chIFITM* locus among Aseel and Kadaknath chicken breeds. The presence of SNVs in both coding and non-coding regions suggests potential functional implications, as variations in non-coding regions can also affect gene expression and regulation. The differences observed between the two reference genomes indicated breed-specific genetic variations within the *chIFITM* locus. According to the published data, the chicken genome architecture confirms that the gene order in the chicken *IFITM* locus is on the q arm of chromosome 5q, *viz*., B4GALNT4-*chIFITM3*-*chIFITM1*-*chIFITM2*-*chIFITM5*-ATHL1 [27].

#### 4.1.1 *IR-chIFITM* gene variants

The *chIFITM1* gene exhibited the highest number of variants compared to the *chIFITM2* and *chIFITM3* genes. It was located at the LOC422993 locus on chromosome 5q [27,56]. In contrast to the Kadaknath breed, a lower number of variants were present in *chIFITM1* genes of the European breed as only two variants (g.13141G > A and g.14105C > T) and only one variant (g.13141G > A) was present in the commercial chicken breeds [28]. On the other hand, eight SNVs in the *chIFITM1* gene were present in various laboratory cell lines (DF1, CEF, HD11 and DT40) [10]. Among these SNVs, one was a synonymous mutation located in the exon region, while the remaining seven were intronic mutations. The research findings highlight the potential variability in the number and type of variants observed in different chicken populations. It was discovered that two specific SNPs within the chicken *IFITM1* gene exhibited a significant correlation with susceptibility to H7N9 virus disease [32]. A significant two haplotypes rs77537847, and rs11246062 were found in *huIFITM1* of the Korean population with ulcerative colitis [37]. However, no such reports were found till date for the association of SNPs against ND virus infection in chickens.

Next to the *chIFITM1* gene, the *chIFITM2* gene was found to have higher genetic variations in the chicken population. A cytosine insertion was observed in the *chIFITM2* non-coding region of the Assel chicken. In line with our findings, Whitehead also discovered a 3bp (TCC) insertion in 5′ UTR in chicken cell lines [10], and similar insertion resulted in frameshift mutation in the *chIFITM2* exonic region of the indigenous chicken breed Onigbaogbe/Rose [28]. However, varying numbers of SNVs were identified: 3 in European (g.14960G > T) [28], 8 in Commercial [28], 2 in Pirbright [28]. and 10 in indigenous chicken [28], and a total of 13 SNVs in the laboratory chicken cell lines [10]. Additionally, two frameshift mutations were observed in the indigenous chicken breeds as g.16243-16244 TG > T and g.16269A > ACC. This suggests that the *chIFITM2* gene might have a higher level of genetic diversity, and variability in different chicken populations, and it was described that the SNP rs1059091-A/G in the *huIFITM2* gene were highly expressive in the Jurkat E6-1 cell population [57].

The *chIFITM3* gene was situated at the LOC770612 locus on chromosome 5q and flanked at the 5′ end by the B4GALNT4 genes and at the 3′ end by the *chIFITM1* genes [27,28,56]. The *chIFITM3* gene had the least number of variations, one unique SNV in each breed was observed in the untranslated region of exon. Similarly, it was discovered that two SNVs and two INDELs in the HD-1 chicken cell line [10]. Comparatively lower numbers of SNVs were reported as 2 in European (g.18814 T > C, g.18862C > T), 1 in Commercial (g.18862C > T), 1 in Pirbright (g.18862C > T), and 3 in Indigenous (g.18862C > T, g.19572G > A and g.19576-19577 AC > A) chicken breeds [28]. This indicates a lower level of genetic variation or diversity in the *chIFITM3* gene among the chicken populations when compared to the *chIFITM1* gene. In contract, thirteen single nucleotide polymorphisms (SNPs) and one insertion/deletion was present in the *IFITM3* gene of Dekalb White and Ross chicken breeds [12]. It was found that the variants rs12252 [31] and rs6598045 [33,58] within the *huIFITM3* gene have a notable correlation with the severity of IAV infection and mortality in COVID-19. These findings indicate a potential role for genetic variations in the *IFITM3* gene in modulating the susceptibility and severity of the infection. However, up to date, there were no reports indicates an association of such SNVs with chicken diseases.

Overall, the results showed the presence of genetic differences between the Aseel and Kadaknath chicken breeds. The presence of SNVs in coding and non-coding regions of the *chIFITM1*, *chIFITM2* and *chIFITM3* genes might have a significant impact on the expression pattern of these genes and the identified SNVs provide valuable insights into the genetic diversity of these antiviral genes and they may have a potential implication for disease resistance and immunity in chickens.

#### 4.1.2 *chIFITM5* gene variants

The *chIFITM5* gene was located after the *chIFITM2* gene and before *ATHL1* on chromosome 5 [27,56]. Among the identified SNVs in Kadaknath three were found to be new variations. Four SNVs such as g.1556674A > G, g.1556686T > C, g.1556705A > C, and g.1556716T > C were discovered in the non-coding region of the *chIFITM5* gene in all chicken cell lines [10]**.** Whereas two SNVs in European, one in commercial and one in Pirbright chicken breeds were also reported [28]**.** These breed-specific SNPs indicate genetic diversity and variation within the *chIFITM5* gene among different chicken populations. Studies have shown that a single recurring mutation, c.-14C > T [59] and c.-9C > A [60], in the *huIFITM5* gene is linked to Osteogenesis Imperfecta Type V in humans. However, no such findings have been reported for the *chIFITM5* gene in chicken breeds.

### 4.2 Prediction of *IR-chIFITM* protein and comparison

The predicted amino acid sequences were evaluated and identified that there was no structural alteration in these protein sequences among the breeds. This infers that the synonymous mutations observed in *chIFITM1*, coupled with the lack of SNVs in the coding regions of *chIFITM2* and *chIFITM3* genes, may not cause functional change of these proteins between the breeds.

The SNVs primarily occurred in the UTRs and introns of the *chIFITM* genes. The UTRs are non-coding regions of the gene that flank the coding sequence and play a role in regulating gene expression [61]**.** The SNVs in the UTR regions and introns did not alter the amino acid sequence of *chIFITM* proteins. Hence, the observed SNVs likely did not have an impact on the protein's primary structure [62]**.** However, it is important to note that SNVs in UTR regions and introns can still have functional implications. They may affect the regulation of gene expression, mRNA stability, or splicing patterns, which can indirectly influence protein production [61,63]**.**

The transition and transversion changes at codon 14 and 112 of Kadaknath *chIFITM1* results in similar amino acids *viz.,* serine and arginine respectively with respect to that of reference sequence. These amino acids were reported to have a critical involvement in innate and adaptive immunity [64,65]**.** These residual amino acids may be conserved for regulation of these protein functions. In line with our observations, we interestingly identified that 8 of the SNVs were synonymous or silent substitutions, potentially leaving the proteins function unchanged [28]. Whereas, it was found that *IFITM3* contains three lysine amino acids which serve as ubiquitination sites [25]. Overall, the analysis indicated that the *chIFITM* protein sequences in both Kadaknath and Aseel chickens remained largely conserved, with most of the genetic variations occurring in noncoding regions and introns.

### 4.3 *In ovo embryonic chIFITM* gene expression against NDV

*In ovo* study revealed several significant findings regarding the impact of various factors on immune gene expression in chicken embryos against NDV. First, the breed factor was found to have a significant influence on immune gene expression and the findings highlight the intricate interplay among genetic factors, tissue types and developmental time points in shaping the immune gene expression response to NDV infection in chicken embryos. Similar kind of study had been carried out for gene expression in ducks and chickens [40], Goosling [57] and RIR chicken [10]**.**

#### 4.3.1 *IR-chIFITM* gene expression

In comparison to the control uninfected embryo of the Aseel and Kadaknath chicken breeds at various times of post-infection, a general trend of gene upregulation in all organs of infected chicken embryos was found. A significant and plentiful amount of *chIFITM1* mRNA was detected in the cecal tonsils of both breeds and in bursa of Aseel. Similarly gene expression pattern was studied in various organs such as bursa [24] and cecal tonsil of RIR chicken against Lyssa virus [24] and lung tissue of duck against IAV [40], cecal tissue of gosling against Tembusu virus [25], intestine tissues of RIR against H9N2 Influenza Viruses [10] and lung of transgenic chicken H5N1 Influenza Viruses [14]**.** In concordance to our study, *chIFITM2* gene expression was specifically observed in all tissues of RIR chicken [56], lung tissue of duck [24,40] and in lung and liver of RIR [10].

In the current study, the *chIFITM3* gene consistently exhibited higher expression levels in the bursa of Aseel chickens compared to all other organs, while a stable expression was noticed in Kadaknath breed. Similar expression was noticed in the thymus, spleen, bursa of Fabricius, cecal tonsil, gastrointestinal tract, trachea, bone marrow, brain, muscle, heart, liver, kidney, lung, and skin of Rhode Island Red (RIR) chickens against Lyssaviruses [56]**,** lung tissue of duck against Influenza virus [24,40]**,** in pulmonary epithelium of human against Human Influenza Virus [66], in respiratory organs of gosling in Tembusu Virus Infection [25]. In addition, the bursa of yellow feathered broilers also expressed strongly *chIFITM3* gene against Avian Reo virus [17] and lung and liver tissues of RIR against H9N2 Influenza Viruses [10]. Apart from the viral infection studies, it was also noticed that heightened expression of the *IFITM3* gene in the spleen and lungs of swine during an inflammatory response induced by lipopolysaccharide (LPS) [67]. The bursa serves as a primary immune organ, while the spleen and cecal tonsil function as secondary immune organs. On the other hand, the lung and heart are vital organs responsible for essential physiological processes. These varying roles and functions among the tissues may contribute to the observed differences in gene expression patterns. It suggests that the expression of *chIFITM* genes may be tissue specific [26,68] and vary across different chicken breeds.

Overall, these results demonstrate that the expression patterns of *IR*-*chIFITM* genes vary across different tissues and time points in response to NDV infection in Aseel and Kadaknath chicken breeds. The observed differences highlight the complex and dynamic nature of the immune response to viral infections in chickens.

#### 4.3.2 *chIFITM5* gene expression

The *chIFITM5* gene exhibited significant upregulation throughout all time points, in bursa, cecal tonsils and heart. However, in the lung, the *chIFITM5* gene did not show substantial up-regulation, displaying low fold changes. On the other hand, in Kadaknath chickens, the *chIFITM5* gene exhibited strong expression specifically in the heart tissue. The gene transcribed in the majority of tissues and were activated specifically in response to infections [69]. The *IFITM5* gene was known for bone-specific expression [70], which was also upregulated in response to the NDV infection in various tissues. Similarly, a significant upregulation of *IFITM5* genes in duck lung tissue was noticed following highly pathogenic IAV infection [24]. The expression levels of *IFITM5* were found to be increased by approximately 5-fold at one day post infection, indicating the induction of these genes during the infection [24].

In contrast to our result, Whitehead reported that there was no significant upregulation of the *chIFITM5* gene observed at any time points of post-infection in the lung tissue of RIR [10]. However, significantly high levels of *chIFITM5* expression were noticed in the liver at 24 and 48 hpi and the intestine at 24 hpi [10]. In contrast, it was observed that *chIFITM5* expression levels were not consistent across any of the tissues [71]. These findings suggest that the regulation and function of the *chIFITM5* gene may vary between different tissues and chicken breeds, potentially playing distinct roles in development, immune response or other biological processes specific to each tissue and breed.

The highest upregulation of *chIFITM* genes was observed at specific time points in each tissue, but there were still notable up-regulations observed at other time points as well. It is important to note that the expression patterns of *chIFITM* genes appeared to be both tissue-specific and breed-specific [10]. Indeed, it is still unclear if certain tissues are able to induce *chIFITM* expression to a greater or lesser extent and whether this is temporally regulated as the embryo develops. The relationship between tissue-specific induction of *chIFITM* gene expression and sequential regulation during embryonic development remains to be elucidated. Further studies are essential to ascertain the relative capabilities of distinct tissues to modulate *chIFITM* gene expression and to differentiate how this regulatory mechanism evolves throughout embryogenesis.

#### 4.3.3 *IFN γ and Mx* gene expression

The findings of our study revealed that the *IFN γ* gene exhibited consistent, and significant upregulation in all of the tissue samples from both Aseel and Kadaknath breeds. However, there were some notable differences between the breeds and tissues. The findings of the studies are consistent with our research regarding the production of *IFN γ* in chickens infected with virulent NDV. A very high level of *IFN γ* expression was noticed in the lymphoid tissues such as thymus, bursa, cecal tonsils, and spleen of NDV infected chickens [[72], [73], [74], [75]]. Taken together, these findings provide further support for the induction of *IFN γ* in various lymphoid tissues in response to NDV infection, highlighting the dynamic nature of the immune response in different organs and stages of infection.

Similarly, the observation was made that the *Mx* gene exhibited high expression levels in the bursa and spleen of Kadaknath chicken embryos. Our findings align with the results presented in Whitehead study, where significant upregulation of the *Mx* gene was observed in the lung, liver tissues, and intestine of Rhode Island Red chicken embryos following *H9N2* infection [10].

These findings indicate that both the *IFN γ* and *Mx* genes play a critical role in the immune response of chickens, as their expression levels were upregulated in multiple tissues. The upregulation of these genes suggests activation of the immune system, possibly in response to an infectious agent or other immune stimuli. Further investigation and functional studies were needed to better understand the specific roles of these genes in chicken immunity and their potential implications for disease resistance in the Aseel and Kadaknath breeds.

## 5 Conclusion

The investigation focused on establishing a unique dataset of SNVs and INDEL for the *chIFITM* locus specific to Aseel and Kadaknath chicken breeds, and the analysis revealed that *chIFITM1* had the most SNVs, followed by *chIFITM2*, with *chIFITM3* exhibiting the least variations. However, the presence of SNVs in *IR-chIFITM* genes of Aseel and Kadaknath indicates substantial genetic diversity in these chicken populations. Despite this diversity, the coding sequences of all *chIFITM* genes exhibit a high degree of conservation, indicating a robust preservation of genetic information. Genetic variations in *chIFITM* genes could impact gene regulation, splicing, and various aspects of gene expression, mRNA stability, and translation efficiency. The resilient and consistent expression of the *chIFITM3* gene across diverse tissues provides compelling support for its potent antiviral efficacy against viral infections. The results, suggests the need for further in-vitro, and in-vivo association studies to understand disease resistance and antiviral protein expression, especially in the context of UTR variants in *chIFITM3* (g.1814938G > A and g.1815386A > C), for effective breeding strategies.

## Data availability statement

The original contributions presented in the study will be provided on request.

## Ethics statement

The experiment was conducted with the approval of the Institutional Bio-safety Committee (IBSC) (Approval Lr. No. 1764/VCRI-NKL/IBSC/2022 dated May 11, 2022 of the Dean, VCRI, Namakkal) and Institutional Animal Ethical committee (IAEC) (Project proposal No. 09/VCRI-NKL/2023 dated September 08, 2023) of TANUVAS-Veterinary College and Research Institute, Namakkal, Tamil Nadu, India.

## Funding

The financial assistance for this research was provided by the Tamil Nadu Veterinary and Animal Sciences University (TANUVAS) in Chennai, Tamil Nadu, India is greatly appreciated. (USO. No. 20652/A1/2020 and No.273/A1/2021, dated February 26, 2021 of Registrar, TANUVAS, Chennai – 51, India.)

## CRediT authorship contribution statement

**Malarmathi Muthusamy:** Writing – review & editing, Writing – original draft, Project administration, Methodology, Formal analysis, Data curation, Conceptualization. **Murali Nagarajan:** Writing – review & editing, Project administration, Conceptualization. **Sivakumar Karuppusamy:** Writing – review & editing, Supervision, Project administration. **Kannaki T. Ramasamy:** Writing – review & editing, Resources, Methodology. **Amutha Ramasamy:** Writing – review & editing, Resources, Project administration. **Ramya Kalaivanan:** Writing – review & editing, Resources, Project administration. **Gopala Krishna Murthy Thippicettipalayam Ramasamy:** Writing – review & editing, Supervision, Methodology, Formal analysis. **Thiruvenkadan Aranganoor Kannan:** Writing – review & editing, Writing – original draft, Supervision, Resources, Project administration, Methodology, Funding acquisition, Conceptualization.

## Declaration of competing interest

The authors declare that they have no known competing financial interests or personal relationships that could have appeared to influence the work reported in this paper.
