# Supplementary material for: “Unveiling the genetic symphony: Diversity and expression of chicken IFITM genes in Aseel and Kadaknath breeds”
Source: Heliyon. 2024 Sep 10;10(18):e37729. doi: 10.1016/j.heliyon.2024.e37729 (PMC11417226; doi:10.1016/j.heliyon.2024.e37729)
Supplement: Multimedia component 1 [file mmc1.docx]

**Supplementary Table 1. *IR-IFITM* genes expression (2^-ΔΔCt^) in Bursa of chicken embryo against NDV**

| **Breed** | **Time Point** | ***chIFITM1*** | ***chIFITM2*** | ***chIFITM3*** |
| --- | --- | --- | --- | --- |
| **Aseel** | **3 hpi** | 4.37 ± 0.18^c^ | 2.53 ± 0.15^a^ | 10.22 ± 0.30^a^ |
|  | **6 hpi** | 3.00 ± 0.05^d^ | 2.07 ± 0.04^ab^ | 10.23 ± 0.19^a^ |
|  | **12 hpi** | 6.04 ± 0.15^b^ | 1.93 ± 0.16^b^ | 9.83 ± 0.11^a^ |
|  | **24 hpi** | 7.61 ± 0.04^a^ | 1.97 ± 0.10^b^ | 9.95 ± 0.15^a^ |
|  | **48 hpi** | 1.67 ± 0.24^c^ | 2.92 ± 0.55^a^ | 9.98 ± 0.62^a^ |
| **Kadaknath** | **3 hpi** | 0.32 ± 0.10^c^ | 3.29 ± 0.62^a^ | 3.86 ± 0.74^a^ |
|  | **6 hpi** | 2.79 ± 0.13^a^ | 2.30 ± 0.38^ab^ | 4.04 ± 1.38^a^ |
|  | **12 hpi** | 1.46 ± 0.09^b^ | 1.16 ± 0.33^b^ | 3.36 ± 0.40^a^ |
|  | **24 hpi** | 2.49 ± 0.17^a^ | 0.40 ± 0.18^c^ | 4.15 ± 0.34^a^ |
|  | **48 hpi** | 1.67 ± 0.06^b^ | 0.61 ± 0.14^c^ | 1.51 ± 0.22^b^ |

Means with different superscripts showed significant differences at p<0.05 within a breed.

**Supplementary Table 2. *IR-IFITM* genes expression (2^-ΔΔCt^) in cecal tonsils of chicken embryo against NDV**

| **Breed** | **Time Point** | ***chIFITM1*** | ***chIFITM2*** | ***chIFITM3*** |
| --- | --- | --- | --- | --- |
| **Aseel** | **3 hpi** | 1.81 ± 0.27^c^ | 1.69 ± 0.13^a^ | 2.97 ± 0.40^a^ |
|  | **6 hpi** | 6.41 ± 2.66^ab^ | 1.13 ± 0.02^b^ | 2.54 ± 0.04^a^ |
|  | **12 hpi** | 9.47 ± 0.63^a^ | 0.63 ± 0.22^b^ | 1.19 ± 0.20^b^ |
|  | **24 hpi** | 1.72 ± 0.38^c^ | 0.98 ± 0.09^b^ | 1.61 ± 0.10^b^ |
|  | **48 hpi** | 2.64 ± 1.02^bc^ | 1.08 ± 0.14^b^ | 1.59 ± 0.34^b^ |
| **Kadaknath** | **3 hpi** | 3.95 ± 0.14^bc^ | 2.11 ± 0.12^a^ | 1.31 ± 0.01^b^ |
|  | **6 hpi** | 5.39 ± 1.08^b^ | 2.42 ± 0.66^a^ | 0.34 ± 0.16^b^ |
|  | **12 hpi** | 5.98 ± 1.22^ab^ | 1.37 ± 0.30^b^ | 1.55 ± 0.27^b^ |
|  | **24 hpi** | 8.67 ± 1.13^a^ | 1.21 ± 0.35^b^ | 5.69 ± 1.75^a^ |
|  | **48 hpi** | 2.13 ± 0.27^c^ | 0.85 ± 0.14^b^ | 0.47 ± 0.15^b^ |

Means with different superscripts showed significant differences at p<0.05 within a breed.

**Supplementary Table 3. *IR-IFITM* genes expression (2^-ΔΔCt^) in heart of chicken embryos against NDV**

| **Breed** | **Time Point** | ***chIFITM1*** | ***chIFITM2*** | ***chIFITM3*** |
| --- | --- | --- | --- | --- |
| **Aseel** | **3 hpi** | ND | 2.33 ± 0.03^bc^ | 2.13 ± 0.08^a^ |
|  | **6 hpi** | ND | 1.89 ± 0.05^c^ | 3.29 ± 0.08^a^ |
|  | **12 hpi** | ND | 2.32 ± 0.07^bc^ | 3.22 ± 0.04^a^ |
|  | **24 hpi** | ND | 3.17 ± 0.15^a^ | 2.53 ± 0.62^a^ |
|  | **48 hpi** | ND | 2.73 ± 0.44^ab^ | 2.84 ± 0.73^a^ |
| **Kadaknath** | **3 hpi** | ND | 2.89 ± 1.67^b^ | 2.57 ± 1.48^a^ |
|  | **6 hpi** | ND | 2.73 ± 1.57^b^ | 2.25 ± 1.30^a^ |
|  | **12 hpi** | ND | 1.51 ± 0.87^b^ | 2.67 ± 1.54^a^ |
|  | **24 hpi** | ND | 10.85 ± 6.26^a^ | 2.08 ± 1.20^a^ |
|  | **48 hpi** | ND | 11.03 ± 6.37^a^ | 2.70 ± 1.56^a^ |

Means with different superscripts showed significant differences at p<0.05 within a breed.

**Supplementary Table 4. *IR-IFITM* genes expression (2^-ΔΔCt^) in lung of chicken embryos against NDV**

| **Breed** | **Time Point** | ***chIFITM1*** | ***chIFITM2*** | ***chIFITM3*** |
| --- | --- | --- | --- | --- |
| **Aseel** | **3 hpi** | 1.41 ± 0.06^d^ | 1.60 ± 0.08^b^ | 3.33 ± 0.09^a^ |
|  | **6 hpi** | 6.05 ± 0.01^a^ | 1.38 ± 0.16^b^ | 3.79 ± 0.80^a^ |
|  | **12 hpi** | 2.40 ± 0.11^c^ | 1.20 ± 0.11^b^ | 3.09 ± 0.11^a^ |
|  | **24 hpi** | 3.13 ± 0.02^b^ | 6.40 ± 0.63^a^ | 4.10 ± 0.63^a^ |
|  | **48 hpi** | 1.46 ± 0.02^d^ | 1.32 ± 0.12^b^ | 3.10 ± 0.02^a^ |
| **Kadaknath** | **3 hpi** | 0.17 ± 0.06^c^ | 17.91 ± 1.12^a^ | 2.81 ± 0.43^a^ |
|  | **6 hpi** | 0.11 ± 0.07^c^ | 17.64 ± 2.14^a^ | 3.10 ± 0.20^a^ |
|  | **12 hpi** | 0.36 ± 0.20^c^ | 12.04 ± 0.36^b^ | 3.17 ± 0.98^a^ |
|  | **24 hpi** | 4.22 ± 1.07^b^ | 9.94 ± 0.69^bc^ | 1.20 ± 0.09^b^ |
|  | **48 hpi** | 6.17 ± 0.46^a^ | 5.23 ± 2.35^c^ | 1.53 ± 0.11^b^ |

Means with different superscripts showed significant differences at p<0.05 within a breed.

**Supplementary Table 5. *IR-IFITM* genes expression (2^-ΔΔCt^) in spleen of chicken embryos against NDV**

| **Breed** | **Time Point** | ***chIFITM1*** | ***chIFITM2*** | ***chIFITM3*** |
| --- | --- | --- | --- | --- |
| **Aseel** | **3 hpi** | ND | 2.17 ± 0.22^b^ | 2.87 ± 0.29 ^ab^ |
|  | **6 hpi** | ND | 2.27 ± 0.03^b^ | 2.09 ± 0.28 ^b^ |
|  | **12 hpi** | ND | 3.51 ± 0.06^a^ | 2.15 ± 0.02 ^b^ |
|  | **24 hpi** | ND | 1.36 ± 0.03^c^ | 3.13 ± 0.14 ^a^ |
|  | **48 hpi** | ND | 1.66 ± 0.01^c^ | 3.53 ± 0.07 ^a^ |
| **Kadaknath** | **3 hpi** | ND | 2.21 ± 0.23^b^ | 1.89 ± 0.16^a^ |
|  | **6 hpi** | ND | 3.77 ± 0.26^a^ | 2.04 ± 0.43^a^ |
|  | **12 hpi** | ND | 4.08 ± 0.21^a^ | 2.33 ± 0.33^a^ |
|  | **24 hpi** | ND | 3.62 ± 0.27 ^a^ | 1.70 ± 0.24^a^ |
|  | **48 hpi** | ND | 1.49 ± 0.10^c^ | 2.18 ± 0.19^a^ |

Means with different superscripts showed significant differences at p<0.05 within a breed.

**Supplementary Table 6. *chIFITM5* genes expression (2^-ΔΔCt^) in various tissues of chicken embryo against NDV**

| **Breed** | **Time Point** | **Bursa** | **Cecal tonsils** | **Heart** | **Lung** | **Spleen** |
| --- | --- | --- | --- | --- | --- | --- |
| **Aseel** | **3 hpi** | 8.70 ± 0.20 ^a^ | 9.79 ± 0.49^a^ | 7.26 ± 0.03^a^ | 1.64 ± 0.11^b^ | 7.42 ± 0.40^a^ |
|  | **6 hpi** | 6.32 ± 0.05 ^b^ | 9.72 ± 0.23^a^ | 6.52 ± 0.06^a^ | 1.27 ± 0.10^b^ | 4.39 ± 0.77^b^ |
|  | **12 hpi** | 6.80 ± 1.36 ^ab^ | 7.53 ± 0.61^b^ | 3.34 ± 0.70^b^ | 1.80 ± 0.15^b^ | 3.83 ± 0.06^bc^ |
|  | **24 hpi** | 5.47 ± 0.35 ^b^ | 9.53 ± 0.01^a^ | 7.80 ± 0.11^a^ | 6.02 ± 0.65^a^ | 2.72 ± 0.05^c^ |
|  | **48 hpi** | 6.87 ± 0.65 ^ab^ | 5.86 ± 0.29^c^ | 6.69 ± 1.28^a^ | 2.22 ± 0.27^b^ | 1.48 ± 0.01^d^ |
| **Kadaknath** | **3 hpi** | 1.96 ± 0.18^a^ | 1.84 ± 0.08^a^ | 10.45 ± 6.03^a^ | 2.12 ± 0.16^a^ | 0.46 ± 0.12^c^ |
|  | **6 hpi** | 1.82 ± 0.17^a^ | 0.31 ± 0.13^c^ | 8.90 ± 5.14^a^ | 1.25 ± 0.13^b^ | 3.25 ± 0.11^a^ |
|  | **12 hpi** | 0.55 ± 0.07^b^ | 1.22 ± 0.15^b^ | 8.37 ± 4.83^a^ | 2.03 ± 0.22^a^ | 2.44 ± 0.50^ab^ |
|  | **24 hpi** | 0.71 ± 0.11^b^ | 0.64 ± 0.20^c^ | 9.46 ± 5.46^a^ | 0.69 ± 0.18^c^ | 1.70 ± 0.33^b^ |
|  | **48 hpi** | 0.43 ± 0.03^b^ | 0.49 ± 0.07^c^ | 10.42 ± 6.02^a^ | 0.47 ± 0.04^c^ | 2.45 ± 0.49^ab^ |

Means with different superscripts showed significant differences at p<0.05 within a breed.

**Supplementary Table 7. *IFN-γ* genes expression (2^-ΔΔCt^) in various tissues of chicken embryo against NDV**

| **Breed** | **Time Point** | **Bursa** | **Cecal tonsils** | **Heart** | **Lung** | **Spleen** |
| --- | --- | --- | --- | --- | --- | --- |
| **Aseel** | **3 hpi** | 7.36 ± 0.62^a^ | 3.48 ± 0.16^a^ | 1.51 ± 0.02^a^ | 2.75 ± 0.05^a^ | 1.79 ± 0.21^b^ |
|  | **6 hpi** | 8.09 ± 0.05^a^ | 4.35 ± 0.15^a^ | 1.49 ± 0.03^a^ | 2.57 ± 0.13^a^ | 1.71 ± 0.14^b^ |
|  | **12 hpi** | 7.32 ± 0.16^a^ | 2.32 ± 0.43^b^ | 1.90 ± 0.01^a^ | 1.49 ± 0.20^c^ | 1.27 ± 0.10^c^ |
|  | **24 hpi** | 6.96 ± 0.02^a^ | 3.55 ± 0.17^a^ | 2.17 ± 0.45^a^ | 2.08 ± 0.11^b^ | 2.17 ± 0.08^a^ |
|  | **48 hpi** | 7.50 ± 0.97^a^ | 0.06 ± 0.01^c^ | 1.74 ± 0.37^a^ | 1.90 ± 0.03^b^ | 2.47 ± 0.01^a^ |
| **Kadaknath** | **3 hpi** | 2.76 ± 0.45^ab^ | 4.47 ± 0.99^b^ | 4.50 ± 2.60^a^ | 0.58 ± 0.17^c^ | 3.00 ± 0.62^a^ |
|  | **6 hpi** | 2.59 ± 0.10^b^ | 4.82 ± 0.64^b^ | 5.49 ± 3.17^a^ | 1.98 ± 0.12^ab^ | 1.33 ± 0.02^b^ |
|  | **12 hpi** | 1.54 ± 0.16^b^ | 9.36 ± 0.67^a^ | 2.48 ± 1.43^ab^ | 1.59 ± 0.06^b^ | 1.40 ± 0.04^b^ |
|  | **24 hpi** | 2.01 ± 0.33^b^ | 7.82 ± 1.07^a^ | 1.14 ± 0.66^b^ | 1.42 ± 0.20^b^ | 1.60 ± 0.25^b^ |
|  | **48 hpi** | 4.29 ± 0.92^a^ | 1.50 ± 0.34^c^ | 4.35 ± 2.5^a^ | 2.51 ± 0.23^a^ | 1.39 ± 0.11^b^ |

Means with different superscripts showed significant differences at p<0.05 within a breed.

**Supplementary Table 8. *Mx* genes expression (2^-ΔΔCt^) in various tissues of chicken embryo against NDV**

| **Breed** | **Time Point** | **Bursa** | **Cecal tonsils** | **Heart** | **Lung** | **Spleen** |
| --- | --- | --- | --- | --- | --- | --- |
| **Aseel** | **3 hpi** | 5.55 ± 0.58^a^ | 2.27 ± 0.04^b^ | 1.16 ± 0.06^b^ | 2.31 ± 0.59 ^bc^ | 2.58 ± 0.49^a^ |
|  | **6 hpi** | 5.94 ± 0.04^a^ | 1.56 ± 0.29^b^ | 1.36 ± 0.13^ab^ | 3.69 ± 0.35 ^ab^ | 1.54 ± 0.09^b^ |
|  | **12 hpi** | 4.63 ± 0.64^a^ | 1.79 ± 0.28^b^ | 2.62 ± 0.42^a^ | 4.25 ± 0.66^a^ | 1.20 ± 0.05^b^ |
|  | **24 hpi** | 6.23 ± 0.06^a^ | 1.61 ± 0.23^b^ | 2.62 ± 0.80^a^ | 1.58 ± 0.22^c^ | 1.74 ± 0.10^b^ |
|  | **48 hpi** | 4.56 ± 0.67^a^ | 1.54 ± 0.20^b^ | 1.23 ± 0.12^b^ | 1.38 ± 0.21^c^ | 3.26 ± 0.23^a^ |
| **Kadaknath** | **3 hpi** | 0.64 ± 0.22^b^ | 0.98 ± 0.36^b^ | 3.84 ± 2.21^a^ | 3.28 ± 0.59^b^ | 7.77 ± 0.43^a^ |
|  | **6 hpi** | 0.95 ± 0.08^b^ | 1.05 ± 0.27^b^ | 4.86 ± 2.80^a^ | 2.58 ± 0.36^b^ | 5.27 ± 0.15^b^ |
|  | **12 hpi** | 5.20 ± 3.00^a^ | 1.21 ± 0.53^b^ | 2.43 ± 1.40^ab^ | 2.15 ± 0.19^b^ | 6.54 ± 0.21^ab^ |
|  | **24 hpi** | 10.68 ± 1.89^a^ | 6.75 ± 0.96^a^ | 1.36 ± 0.78^b^ | 6.95 ± 1.37^a^ | 6.69 ± 0.63 ^ab^ |
|  | **48 hpi** | 7.85 ± 1.32^a^ | 1.87 ± 0.22^b^ | 1.33 ± 0.77^b^ | 2.37 ± 0.31^b^ | 6.56 ± 0.59 ^ab^ |

Means with different superscripts showed significant differences at p<0.05 within a breed.
